# The impact of JUUL market entry on cigarette sales: evidence from a major chain retailer in Canada

**DOI:** 10.1186/s12954-023-00790-1

**Published:** 2023-05-09

**Authors:** Yingying Xu, Anindya Sen, Tengjiao Chen, Christopher M. Harris, Shivaani Prakash

**Affiliations:** 1 JUUL Labs, Inc., Washington, DC, USA; 2 Department of Economics, University of Waterloo, Waterloo, Canada

**Keywords:** Electronic nicotine delivery systems, JUUL, Market entry, Cigarette and vaping product sales in Canada

## Abstract

**Background:**

Electronic nicotine delivery systems (ENDS), such as the JUUL system, are nicotine products for adults who currently smoke cigarettes but are looking for an alternative to combustible cigarettes. Sales of ENDS products were legislatively acknowledged and authorized federally in Canada with the Royal Assent of the Tobacco and Vaping Products Act in 2018.

**Methods:**

With the unique dataset from a major chain retailer in Canada, we evaluated the impacts of JUUL market entry on cigarette sales across Canada from January 2017 to August 2019 using two-way fixed effects panel regression models by leveraging on the entry time variation at the city level. We conducted various robustness checks and a permutation test to validate our results.

**Results:**

Our estimates suggested that JUUL market entry was, on average, significantly correlated with a 1.65% per-month decrease in cigarette sales during the initial months, and with a potentially larger impact on urban areas. Our results were robust across various specifications and tests. These findings implied that JUUL and combustible cigarettes act as economic substitutes during the study time period in Canada.

**Conclusions:**

These results suggested that local availability of ENDS products, such as JUUL, has the potential to reduce local cigarette consumption.

## Background

Smoking remains the leading cause of preventable death and disease among adults [1]. Electronic nicotine delivery systems (ENDS), also known as e-cigarettes, are alternative nicotine products designed and intended for adults who currently smoke cigarettes to switch completely away from cigarettes by providing nicotine without the smoke from burning tobacco. Specifically, ENDS products are a non-combustible substitute that deliver nicotine through an aerosol.

The National Academies of Science, Engineering and Medicine [2], Public Health England [3] and Health Canada [4] indicated that ENDS products, while not harmless, significantly reduce exposure of adults who smoke combustible cigarettes to toxic and cancer-causing chemicals, and may be a less harmful alternative to smoking [5]. Recent clinical and population-level research suggested that adults who switched completely from combustible tobacco to ENDS use showed significant decreases in biomarkers of exposure to several toxicants [6–8]. Although there is evidence around the harm reduction potential of ENDS, there remains considerable debate regarding the overall population health impact of commercial sale of ENDS products; specifically, some critics state that while ENDS are effective in clinical trials, the increased consumption of ENDS has not been clearly shown to be an effective tobacco control strategy [9–12].

Several studies utilizing public health surveillance data in the US and UK demonstrated an association between increased vaping prevalence and decreased smoking prevalence among adults following introduction of ENDS products [13, 14], as well as an increase in successful quit attempts by people who currently smoke using ENDS [5, 15]. Several other causal inference studies leveraged policy variation affecting availability of ENDS products, such as changes in ENDS taxes [15–17], advertising regulations [18], state-level ENDS sales bans [19–21], or minimum legal age of sale laws [22, 23] to implement a quasi-experimental study design. This body of literature generally demonstrated that restricting ENDS availability leads to increases in cigarette sales or smoking prevalence, offering evidence on the effects of these products as alternatives to combustible cigarettes.

In terms of Canadian specific research, most studies focused on youth and young adult behavior, with limited research on the overall impact of ENDS on cigarette smoking among Canadians [24–33]. Irvine and Nguyen investigated trends in Canadian cigarette sale with a focus on the effects of graphic warning labels [34]. East et al. [35] compared two nationally representative, but methodologically different surveys fielded before and after vaping products were legislatively acknowledged and authorized federally in 2018 and found apparent decreases in combustible cigarette smoking with apparent increases in ENDS use. Another recent study found some suggestive evidence, though not consistent across regions, that cigarette smoking declined faster after ENDS introduction in Canada using the Canadian Tobacco Alcohol and Drugs Survey and cigarette sales data from Health Canada [36].

This study seeks to provide rigorous empirical evidence of the association between ENDS and combustible cigarette sales in Canada by utilizing the time variation of the introduction of the JUUL system (JUUL). The JUUL system is an ENDS product that entered the Canadian market in late 2018 and gained substantial market share since its launch.^1^ This was accomplished through access to a proprietary dataset of cigarette, ENDS products, and JUUL sales from a sample with over 600 convenience stores from a major chain brand in Ontario (ON) and four other provinces from January 2017 to August 2019, covering a period of 32 months. These data allowed us to study the effects of JUUL’s entry into the Canadian market by providing store-level sales data across time. This dataset enabled us to evaluate the correlation between cigarette and vaping sales and the introduction of JUUL products at the city level, controlling for the effects of unobserved provincial and time-specific shocks that might otherwise lead to confounded estimates. Further, the use of actual sales data is a useful contribution given the preponderance of research based on self-reported use of cigarettes and vaping products.

## Methods

### Data

Monthly combustible cigarette and ENDS product sales data at the store level were obtained from one of the largest convenience store chain brands in Canada.^2^ Cigarette sales data were measured in sales volume (by cigarette carton, and 1 carton contains 200 sticks) and sales value (in $CAD) by combining sales from all brands of available cigarettes in stores. To determine the JUUL entry month at the city level, we also aggregated and investigated JUUL’s syndicated sell-through commercial sales data in Canada.

Our data sample consisted of 625 stores in 159 cities and towns. The study period was from January 2017 to August 2019, covering a period of 32 months, including the year prior to and the year following JUUL market entry. The balanced panel, which only included the stores with 32 complete observations, consisted of 603 stores in 154 cities and towns from five provinces including Ontario (ON). Hence, the balanced panel dataset for this study had 19,296 observations (603 stores by 32 months).^3^ Among them, 422 stores were located in large metropolitan areas, for example, Toronto, ON. A presence across these provinces and major metropolitan areas implied coverage of a significant percentage of the Canadian population.

**Fig. 1:**
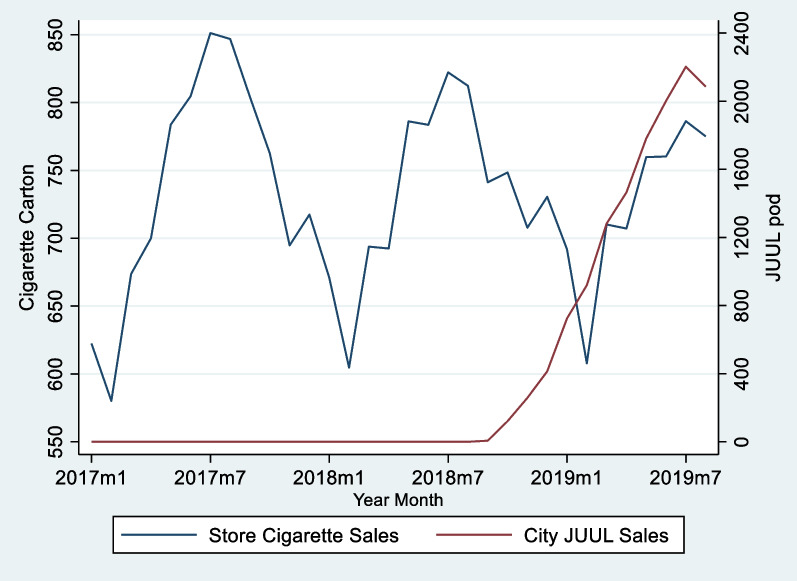
Store-level Cigarettes and City-level JUUL Sales, January 2017 to August 2019 (All Stores). *Note* the cigarette sales volume is the average sales in 603 stores; the JUUL sales volume is the average sales in 154 localities. The dataset included 603 stores in 154 cities and towns from five provinces including Ontario. 422 stores are located in large urban areas. The time period for this study is January 2017 to August 2019

Figure 1 shows the average cigarette sales and the initial growth of JUUL sales after its market entry. The figure demonstrates the seasonal variation in cigarette sales. There was a clear decline in peak cigarette sales volume over time with a drop that occurred after JUUL entry. However, JUUL entry could have coincided with an overall declining trend in cigarette sales. Hence, assessing the sensitivity of econometric findings with time-specific trends becomes important, and this is a strategy we employed, as discussed in detail below.

**Fig. 2:**
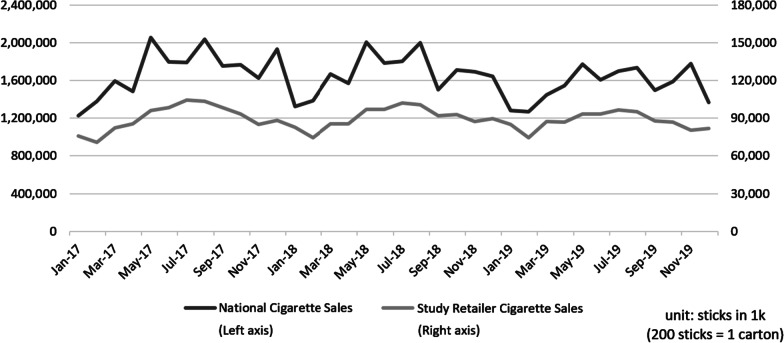
National Cigarette Sales versus Retailer Cigarette Sales. *Note* national cigarette sales volume was obtained from Statistics Canada (Table 16-10-0044-01). The cigarette sales volume in the study retailer was aggregated from the balanced panel with sales volume in 603 stores. All volumes were measured by cigarette stick (× 1000). The dataset included 603 stores in 154 cities and towns from five provinces including Ontario

Figure 2 demonstrates that these trends were consistent and highly correlated with overall cigarette sales in Canada from Statistics Canada data [37], providing reassurance on the reliability of our store-level data sample. The correlation between national cigarette sales and the store-level data is important given the fact that our retailer sample was not from a random selection, which may differ from overall city-level impact of market entry if the retailer is not representative. For cigarette sales, we observed no indication that this specific retailer sample was systematically different from other tobacco product retailers, and the cigarette sales of the retailers in the study followed the same overall trend observed from national data.

JUUL entry date was defined by the first month that JUUL sales were reported in each city/town in syndicated commercial data; this date was used to delineate the pre- and post-entry period for all stores within the city. The earliest entry date following JUUL country launch was September 2018. The JUUL entry date was determined by availability of JUUL at the city level, rather than availability of JUUL at the store level, since our focus is on the impact of availability of JUUL in local markets. Table 1 summarizes the entry month of JUUL in different cities and towns. Depending on the specific market, entry in most provinces occurred during September or October 2018, and a few more happened during the early part of 2019.

**Table 1:** JUUL entry month at the city level

Month of JUUL entry	ON	Province A	Province B	Province C	Province D	Total
Sept 2018	18	28	6	1	4	57
Oct 2018	3	11	39	3	7	63
Nov 2018	0	0	6	1	0	7
Dec 2018	1	0	1	1	0	3
Jan 2019	5	5	1	1	4	16
Feb 2019	0	1	4	0	1	6
Mar 2019	0	1	1	0	0	2
Total	27	46	58	7	16	154

The dataset included 603 stores in 154 cities and towns from five provinces including Ontario. 422 stores are located in large urban areas, for instance, Toronto, ON

**Fig. 3:**
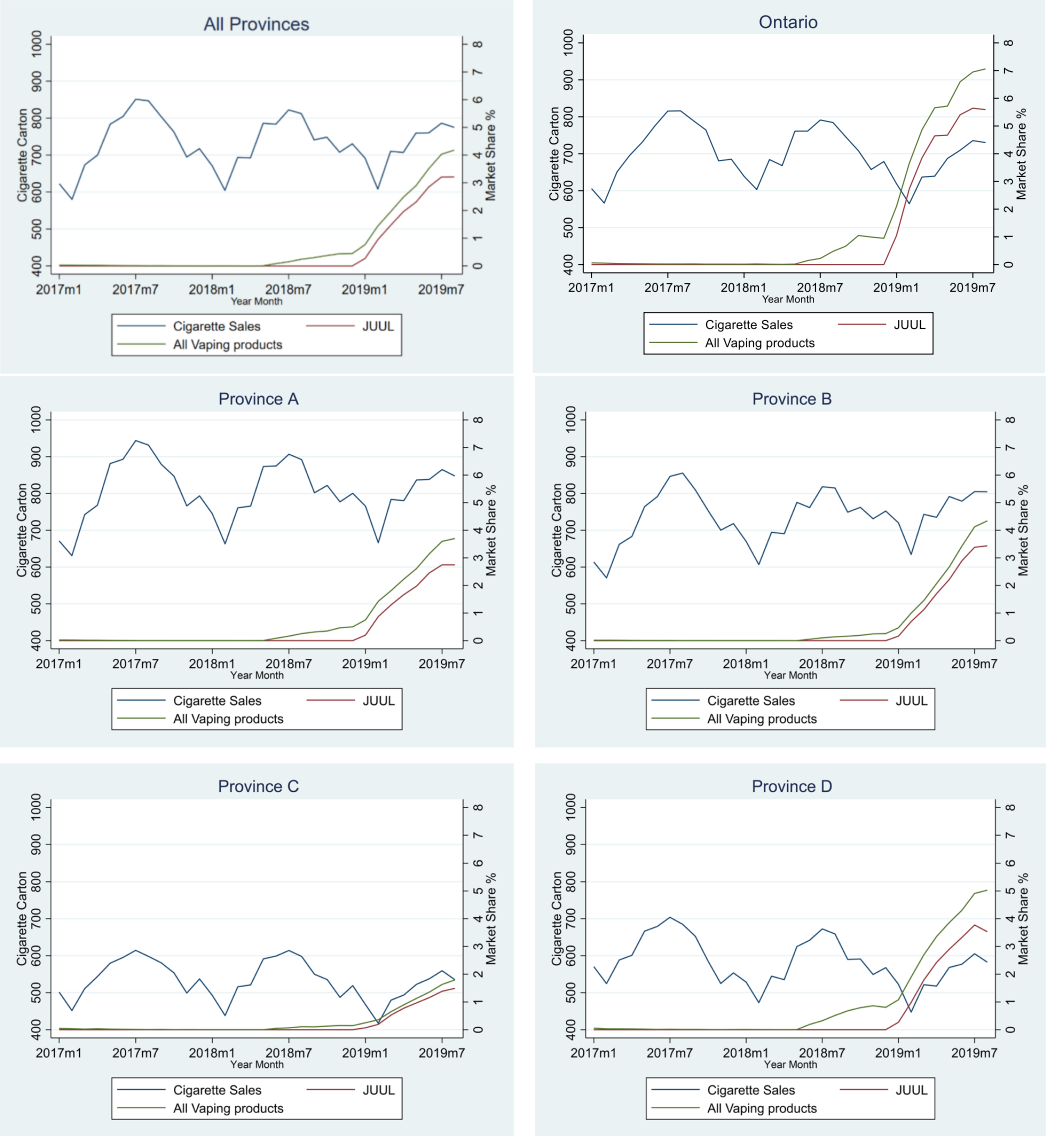
Store-level Cigarettes Sales and JUUL Market Share (Separated by Province). *Note* the cigarette sales volume and market shares are the average in 603 stores used in this study. The indicators for market share are the value of vaping products or JUUL sales in dollars as percentages of total amount of total cigarettes and ENDS products sales. The time period for this study is January 2017 to August 2019

Figure 3 yields some insights on the growth and proportion of JUUL products among total ENDS products across provinces. Specifically, based on our sample from the chain retailer, JUUL products represented about 70% to 80% of all vaping sales by the end of the study period in each province. When examined as a percentage of total sales of combustible cigarettes and vaping products, JUUL market share was relatively high in Ontario (roughly 7%) and as low as approximately 2% in two other provinces. Despite the differences in magnitude across regions, the initial growth trends after the JUUL entry were all almost linear, at least for the first five to eight months.

### Empirical Strategy

The following panel regression model was utilized to estimate the impact of the market entry and initial growth of JUUL on cigarette sales by leveraging on the entry time variation at the city level.

1 $$Y_{sct} = \alpha_{s} + \delta E_{ct} + x_{ct}^{^{\prime}} \beta + \gamma_{t} + \varepsilon_{sct} ,$$

where ${Y}_{sct}$ is the dependent variable, which can be cigarette volume or value sales (or log-transformed cigarette sales) in store $s$, located in city $c$, in month $t$. Market entry variable, $E_{ct}$, can be a binary variable for indicating whether JUUL was available for purchase in the local city $s$ and is set to one if the observations were in the post-entry period, and zero if in the pre-entry period. In addition to using 0/1 dummy variables for the city-level entry, we also constructed a linear-trend variable to simulate city-level JUUL sales after the market entry over time during the post-entry period as $E_{ct} = \left( {t + 1 - \epsilon_{c} } \right)*1\left( {t \ge \epsilon_{c} } \right)$, where $\epsilon_{c}$ was the first month in city $c$ when JUUL became available. All stores in the sample offered JUUL products at some point during the study period. We included store fixed effects as $\alpha_{s}$ to capture time-invariant heterogeneity of stores, and year-month fixed effects $\gamma_{t}$ to absorb overall trends and shocks in cigarette sales over time; such as seasonal variation, population-level tobacco use patterns, and the decline trend in cigarette sales nationwide. There were no changes in ENDS taxes during the study period. We also controlled for average monthly local temperature (data from nearest weather stations based on store locations), province-level CPI, unemployment rate, and gasoline price through covariate matrix $x_{ct}$. For stores located in urban areas with available metropolitan-level demographics, we used metropolitan-level measures of these control variables instead. Summary statistics of selected variables are provided in Table 2.

**Table 2:** Descriptive Statistics

	Range	Mean	Std. Dev.
Cigarette sales volume (Carton)	(76.2, 2549.3)	732.3	297.4
Cigarette sales value (CAN$)	(8314, 284,347)	84,892	34,033
Cigarette price	(80.2, 136.1)	116.4	9.1
JUUL city entry (0/1)	(0, 1)	0.356	0.479
JUUL market share (0–1)	(0, 0.384)	0.00733	0.0178
Temperature (C)	(− 25.6, 25.1)	6.53	10.5
CPI	(121.6, 144.1)	134.9	5.5
Unemployment rate (%)	(3, 10)	6.1	1.4
Gasoline price (cents per liter)	(87.6, 169)	121.3	19.5

Data are based on the balanced panel for regressions in Table 3. A total of 19,296 (603 stores × 32 months) data points were used. We used log-transformed measures for cigarette sales as the outcomes in the regressions. For CPI, unemployment rate and gasoline price, we used metropolitan-level demographics for stores in metropolitan areas (urban) and province-level data if metropolitan-level demographics were not available

Our main results are based on the balanced panel dataset with 603 stores. As robustness checks, we ran the model with unbalanced panel on a sample that included 22 additional stores that do not have the full 32-month observations. To see the impact on urban areas, the same model was implemented using 422 stores located in large metropolitan areas (henceforth: urban areas). In terms of sensitivity tests, we ran the model with different settings of time trends and fixed effects besides year-month fixed effects, including province-specific linear time trend, city-specific seasonal control, separated year and month fixed effects, and city-specific linear time trend.

In addition, we performed an event-study analysis, which regressed cigarette sales on lag and lead indicators for the entry of JUUL at the city-level, to map out the pattern of the observed pre-period trends and post-period changes in cigarette sales following JUUL market entry.

To examine that the relationship between JUUL entry and cigarette sales was not due to coincidence, we conducted a permutation test to validate the plausible causal relationship in this model. We randomly reassigned all stores with the JUUL market entry dates recorded in our sample with equal chance, to check whether a relationship still exists between JUUL entry and cigarette sales. After the random permutation process, we ran the same empirical regression and recorded the coefficient of ${E}_{ct}$. We repeated this permutation procedure 1000 times to generate a range of estimated effects and checked to see how likely the estimated effect obtained from the model using real JUUL market entry dates could happen. This test was conducted to ease the concern regarding population-level declines in cigarette sales, and to demonstrate that the relative changes in cigarette sales in the post-JUUL-entry period were not likely driven by coincidence or a correlation with some unobserved time-specific shocks.

## Results

Table 3 reports our main findings from the panel regressions of the impact of city-level JUUL entry on store-level cigarette sales using the model described by Eq. (1). All specifications had store-level fixed effects and year-month fixed effects, along with additional controls as described above. The odd-number columns report the results based on the entire balanced panel, while the even-number columns show those from only the urban area sample. The first four columns estimate coefficients of the binary indicator of city-level JUUL entry with log-transformed cigarette sales as dependent variables in Columns (1) and (2) and level dependent variables in Columns (3) and (4). The models in the last two columns estimated the monthly effect on cigarette sales by assuming linear growth of JUUL sales after its market entry. Robust standard errors for coefficient estimates, in parenthesis, were clustered at the store level. We reported percentage effects in brackets.^4^

**Table 3:** Impact of JUUL Market Entry on Store-level Cigarette Volume sales

Monthly cigarettes volume sales	(1)	(2)	(3)	(4)	(5)	(6)
	Log-dummy entry	Log-dummy entry, Urban	Level-dummy entry	Level-dummy entry, Urban	Level-linear growth	Level-linear growth, Urban
Monthly average (09/2017 to 08/2018)			737.1	725.4	737.1	725.4
City JUUL entry	− 0.0157**	− 0.0259**	− 8.177*	− 14.91***		
	[1.57%]	[2.59%]	[1.11%]	[2.06%]		
	(0.00738)	(0.0103)	(4.545)	(5.368)		
Linear trend after JUUL entry					− 12.15***	− 25.07***
					**[1.65%/month]**	[3.46%/month]
					**(4.143)**	(5.251)
Observations	19,296	13,504	19,296	13,504	19,296	13,504
Adjusted *R*^2^	0.959	0.965	0.950	0.955	0.950	0.956
Store FE	Yes	Yes	Yes	Yes	Yes	Yes
Year-month FE	Yes	Yes	Yes	Yes	Yes	Yes
Other controls	Yes	Yes	Yes	Yes	Yes	Yes

***p < 0.01, **p < 0.05, *p < 0.1

Year-month fixed effects (FE) are unique for each year and month. A total of 32 year-month fixed effects were included in the regressions. Control variables for local temperature, gasoline price, CPI, and unemployment rate were included in the regressions but omitted in the table. Robust errors for coefficient estimates were clustered at the store level. The dataset, used in Columns (1), (3), and (5), included 603 stores in 154 cities and towns from five provinces including Ontario. The urban specifications Columns (2), (4), and (6) included 422 stores that are located in large urban areas, for example Toronto, ON. The analysis used observations from January 2017 to August 2019

We found negative and significant coefficients across all specifications from both JUUL city-level entry and linear trend after JUUL entry. JUUL market entry at the city-level was associated with a decrease in store-level cigarette volume sales by 1.57% (*p* < 0.05) based on Column (1) and an even larger drop of 2.59% in urban areas based on Column (2). We found consistent results from Columns (3) and (4) using a level dependent variable. Note that JUUL products were introduced in most cities in September and October of 2018 and with the year-month fixed effects, the dummy entry indicators at the city-level only capture the effect of the first one to two months. Columns (5) and (6) show that the market entry of JUUL was associated with a 1.65% per-month decrease overall and a 3.46% per-month decrease in urban areas, respectively, during the initial months. Appendix Table 5 summarizes the results using cigarette value sales (in $CAD) as dependent variables, and all the results were consistent and statistically significant except that the estimated magnitudes using value sales are even larger than those from Table 3. Our results suggested that the overall impact of JUUL entry on cigarette sales was probably largely driven by changes in cigarette sales in urban areas. Appendix Table 6 reports the results using the whole dataset with unbalanced observations, and again, the results were very similar to our estimates.

**Fig. 4:**
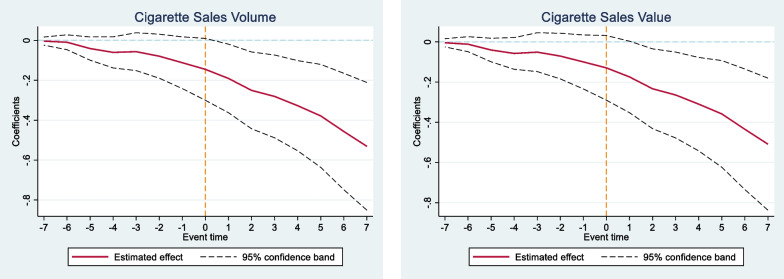
Event Study Analysis for Impact of JUUL Market Entry on Cigarette Sales. *Note* the figure above displays the regression coefficient estimates and two-tailed 95% confidence intervals after controlling for all relevant factors. To map out the pattern, we regress cigarette sales on lag and lead indicators for JUUL market entry at the city level

Figure 4 shows the results of the event study analysis, which presents the estimators on cigarette sales for the seven months preceding and following JUUL market entry date (with each observation in the figure representing the coefficient with confidence intervals for each month pre- and post-entry). We found that prior to JUUL entry in Canadian city-level markets, no significant pre-trend was observed for cigarette sales after controlling for all relevant factors described in the main specification. Significant declines in cigarette sales were observed after JUUL market entry, and we saw an increasing impact in the months following entry date.

Table 4 reports the results of our robustness checks, comparing to the benchmark specification shown in Column (5) of Table 3. To show that our estimates are not sensitive to other control variables, Column (1) in Table 4 provided the estimate with only the store and year-month fixed effects. Column (2) used the same specification as our benchmark one, but with robust errors clustered at the city level. To eliminate the concern that cigarette price changes rather than the introduction of JUUL caused the drop in cigarette sales, the specification in Column (3) further controlled for cigarette price though it could be potentially endogenous. All of these estimates of the JUUL market entry effects were statistically significant at least at the 5% level and remained similar in magnitude as the one from the benchmark.

**Table 4:** Robustness checks

Monthly cigarettes volume sales	(1)	(2)	(3)	(4)	(5)	(6)	(7)
	No other control	Cluster at the city level	w/cig price	w/province-specific linear trend	w/city-specific season control	w/year and month FE separately	w/city-specific linear time trends
Monthly average (09/2017–08/2018)				737.1			
Linear Trend after JUUL Entry	− 14.25***	− 12.15**	− 14.80***	− 10.44**	− 12.01***	− 13.09***	− 10.99***
	(4.073)	(4.660)	(4.264)	(4.125)	(4.236)	(0.860)	(0.596)
Cigarette Price			− 0.00226**				
			(0.000876)				
Observations	19,296	19,296	19,296	19,296	19,296	19,296	19,296
Adjusted *R*^2^	0.950	0.950	0.950	0.952	0.956	0.948	0.957
Store FE	Yes	Yes	Yes	Yes	Yes	Yes	Yes
Year-month FE	Yes	Yes	Yes	Yes	Yes		
Province-specific linear time trend				Yes			
City-specific seasonal control					Yes		
Year FE and month FE						Yes	Yes
City-specific linear time trend							Yes
Robust errors clustered at the city level		Yes					
Other controls		Yes	Yes	Yes	Yes	Yes	Yes

***p < 0.01, **p < 0.05, *p < 0.1

Year-month fixed effects (FE) are unique for each year and month. A total of 32 year-month fixed effects were included in the regressions. Control variables for local temperature, gasoline price, CPI, and unemployment rate were included in the specifications (2) to (5) but omitted in the table. Robust errors for coefficient estimates were clustered at the store level, except column (2). The dataset included 603 stores in 154 cities and towns from five provinces including Ontario. The analysis used observations from January 2017 to August 2019

Specifications in Columns (4) to (7) provide extra robustness checks with different time fixed effects and time trend controls. In addition to year-month fixed effects, the model in Column (4) further controlled for province-specific linear time trends, and city-specific seasonal controls were added to the specification in Column (5). Instead of year-month fixed effects, specifications in Columns (6) and (7) used separated year and month fixed effects, and with city-specific linear time trends shown in Column (7). Although the magnitude of the coefficients varied slightly across these specifications, all of them were consistent with our estimate from the benchmark specification and were statistically significant at least at the 5% level.

**Fig. 5:**
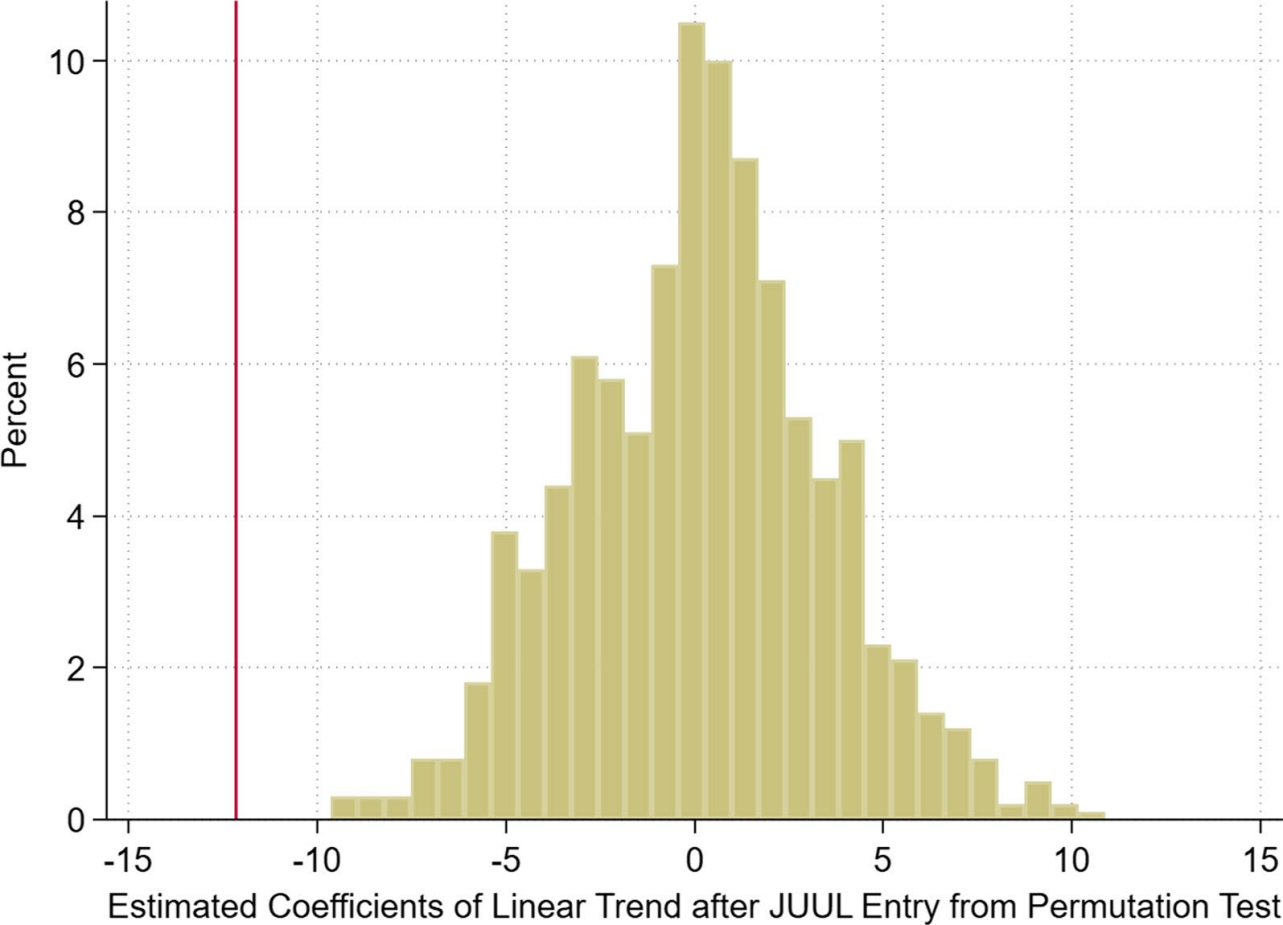
Results of the Permutation Test. *Note* the figure shows the distribution of the estimated coefficients from the permutation test with 1000 repeats, with the red vertical line indicating our preferred estimate at − 12.15 from Column (5) of Table 3

Table 4 suggests that our results were robust across different specifications. Figure 5 illustrates the distribution of the estimated coefficients from our permutation test with 1000 repeats, with the vertical line indicating our preferred estimate at − 12.15 based on Column (5) of Table 3. The permutation test revealed that our estimated effect from the benchmark specification was extremely unlikely to be observed from a random reassignment of JUUL entry dates, as none of the 1000 repeats generated a coefficient with the same negative magnitude. This implied that the impact we captured in the panel regression model regarding the relative decreases in cigarette sales can be better explained by the JUUL market entry than a coincidence or other unobserved events.

## Conclusions

This study utilized monthly panel data from a large convenience chain retailer and the plausible exogenous variation in timing of JUUL city entry to estimate the effects of the market introduction of JUUL products on store-level cigarette sales. We found that the introduction of JUUL significantly correlated with a 1.65% per-month decrease in cigarette sales during the initial months. Assuming the market share of JUUL stabilizes within five to eight months after its introduction, our estimate implies the market entry of JUUL could be associated with a total of 8.3% to 13.2% drop in cigarette sales. Given that there are approximately 3.67 million people who smoke in Canada and 44.7% of people who currently smoke or recently quit had attempted to quit smoking at least once in the past year [35], these results indicate ENDS use could contribute to a potentially large public health impact if abstinence from smoking is maintained long-term. These data extend existing research based on self-reported survey data, and the results of this research offer additional evidence that local availability of ENDS products is associated with reduced cigarette sales in Canada.

From a policy perspective, these results are important, given concerns within tobacco control and public health regarding the possibility that ENDS products might be a significant factor in continued use of combustible cigarettes or the subsequent uptake of cigarette smoking. Instead, these findings support the potential of ENDS leading to partial or complete displacement of smoking cigarettes. We found consistent results from multiple specifications and an array of sensitivity tests. Based on these robustness checks and the permutation test, it is unlikely that an alternative explanation could account for why JUUL market entry in a city is significantly associated with subsequent cigarette sales declines. Our results also suggest that these findings were probably driven largely by declines in urban areas. Given the higher density of tobacco product retailers and people who smoke in urban areas, it seems likely that this is where the availability of alternative products would have a larger impact on consumer behavior that is measurable at the population level.

From a broader perspective, this paper adds to the body of research on changes in the nicotine and tobacco market after the introduction of ENDS products. It supplements previous observational studies that rely on self-reported behavioral survey data and leverages a strong empirical approach along with numerous robustness checks. While many researchers have descriptively reported on changes in adult prevalence of ENDS use and smoking in recent years [35, 38–40], there is still a lack of methodologically rigorous research on the population-level impact of the introduction of ENDS products.

Stoklosa et al. [36, 41] evaluated the impact of entry of another combustible cigarette alternative, IQOS, at the province-level in Japan, by utilizing exogenous variation in product rollout they found that the introduction of IQOS likely reduced cigarette sales in Japan. Similar findings on IQOS entry were reported in trend analyses in Japan by Cummings, Nahhas and Sweanor [42]. Wu et al. examined smoking prevalence and per capita cigarette sales in three countries with various regulatory regimes. They found in Canada, where vaping products were largely unregulated preceding the Tobacco and Vaping Products Act (TVPA), there were some off-trend changes in several provinces [36]. Our research adds to these findings, indicating the broadening of the ENDS marketplace following the TVPA also lead to significant changes in cigarette sales in Canada.

There are several caveats that are worth mentioning when interpreting our results. First, since our dataset only came from a major chain retailer, one limitation of this study is that our estimate may not be able to reflect the entire retail landscape, especially in untracked channels. Moreover, the dataset is not sufficiently granular to identify sales of different nicotine strength or flavors of JUULpods during the study period. Second, we recognize that these results, based upon a proprietary data set and presented largely by current or former JUUL employees, could raise concerns on possible bias in estimation methods and reported results. To mitigate such concerns, Professor Sen, as an independent external scholar, assumed the role of an ‘auditor’, in addition to being largely responsible for developing and choosing the appropriate estimation methodologies. Specifically, given his knowledge of the literature and research expertise, Professor Sen established the econometric models that should be estimated, in consultation with Dr. Xu, Dr. Chen, and Dr. Prakash. Dr. Xu and Dr. Chen wrote the codes, and Professor Sen audited all the coding and the statistical results and econometric estimates to ensure that the analysis was done correctly and free from any intentional bias. The writing of the manuscript was done largely by Professor Sen, Dr. Chen, and Dr. Harris. Hence, Dr. Sen’s role as an ‘auditor’ should yield confidence on the robustness of our findings. Ultimately, the authors hope that more independent researchers will respond with additional research to address the important issue of the relationship between ENDS and cigarettes at the population level. Finally, a number of policies including taxation, restricting on nicotine strength and flavor, as well as marketing and retailer restrictions for ENDS products have been proposed and passed in Canada following the conclusion of this study’s time-period. Future research is needed to assess the effects of these policies on ENDS and cigarette sales.

The net population impact of ENDS products, such as JUUL, cannot be determined solely based on sales changes between ENDS and cigarettes, as it is highly dependent on other factors. However, the findings of this study, which suggest the availability of JUUL is associated with declines in cigarette consumption over time at the population level, are consistent with previous evidence that ENDS products are used by some adults who currently smoke as an alternative to combustible cigarettes and can potentially help adults who currently smoke switch away from smoking [43]. Longer term data are still needed on the relative harm associated with ENDS products use and how this specifically translates to changes in morbidity and mortality risk as compared to smoking. However, the current body of evidence suggests ENDS can provide a potentially less harmful alternative to cigarette smoking [6–8, 44].

Finally, given the specificity of our focus on JUUL market entry within the first year, these findings may not extrapolate to the impact of entry of all ENDS products over time or in other settings, as product characteristics and market conditions could be quite different. More research is still needed on the long-term effects of ENDS availability at the population level, especially in light of changing policy environments. This work only estimates the effect of early-stage market entry of a popular ENDS product. It is also worth noting that the substitution effect that appears in the data was in an environment where switching to vaping was not actively encouraged through risk-proportionate regulation or public information campaigns as, for example, is the case in the United Kingdom^5^ and New Zealand.^6^ Efforts to encourage the use of ENDS may accelerate the displacement of cigarettes. Prior research has found that policies impacting ENDS accessibility such as taxes and bans can lead to increases in cigarette sales [16, 17, 19], and it is possible that the passage of comparable policies after our study period translate to similar effects. However, as noted above, our findings illustrate the impact of ENDS introduction by utilizing plausible exogenous variation in timing of entry of one product specifically as an example. Given that our findings are robust to a number of specifications and causality tests, they collectively demonstrate the potential impact that availability of ENDS products can have on reducing morbidity and mortality associated with smoking cigarettes.

## Data Availability

We are unable to share the sales data used in this study due to the contract restrictions with this retailer, and we have to keep the identity of the retailer anonymous. JUUL Labs, Inc. does not own the data used in this study. Interested parties may contact Michael Fisher (Michael.fisher@juul.com) for additional information regarding these data.

## Appendix

See Tables

**Table 5:** Impact of JUUL Market Entry on Store-level Cigarette Value Sales

Monthly cigarettes value sales	(1)	(2)	(3)	(4)	(5)	(6)
	Log-dummy entry	Log-dummy entry, Urban	Level-dummy entry	Level-dummy entry, Urban	Level-linear growth	Level-linear growth, Urban
Monthly average (09/2017 to 08/2018)			85,419.73	84,846.77	85,419.73	84,846.77
City JUUL entry (0/1)	− 0.0322***	− 0.0382***	− 2341.7***	− 2689.2***		
	(0.00710)	(0.00934)	(518.3)	(562.5)		
Linear growth after JUUL entry					− 2410.0***	− 4435.8***
					(488.8)	(789.0)
Observations	19,296	13,504	19,296	13,504	19,296	13,504
Adjusted *R*^2^	0.958	0.964	0.947	0.952	0.948	0.954
Store FE	Yes	Yes	Yes	Yes	Yes	Yes
Year-month FE	Yes	Yes	Yes	Yes	Yes	Yes
Other controls	Yes	Yes	Yes	Yes	Yes	Yes

****p* < 0.01, ***p* < 0.05, **p* < 0.1

Year-month fixed effects (FE) are unique for each year and month. A total of 32 year-month fixed effects were included in the regressions. Control variables for local temperature, gasoline price, CPI, and unemployment rate were included in the regressions but omitted in the table. Robust errors for coefficient estimates were clustered at the store level. The dataset, used in Columns (1), (3), and (5), included 603 stores in 154 cities and towns from five provinces including Ontario. The urban specifications Columns (2), (4), and (6) included 422 stores that are located in large urban areas, for instance, Toronto, ON. The analysis used observations from January 2017 to August 2019

^5^ and

**Table 6:** Impact of JUUL market entry on store-level cigarette volume sales with unbalanced panel

Monthly cigarettes volume sales	(1)	(2)	(3)	(4)	(5)	(6)
	Log-dummy entry	Log-dummy entry, Urban	Level-dummy entry	Level-dummy entry, Urban	Level-linear growth	Level-linear growth, Urban
Monthly average (09/2017–08/2018)			732.0	719.4	732.0	719.4
City JUUL Entry	− 0.0210**	− 0.0376***	− 9.983**	− 18.56***		
	(0.00840)	(0.0122)	(4.710)	(5.689)		
Linear Growth after JUUL Entry					− 12.86***	− 26.85***
					(4.190)	(5.491)
Observations	19,764	13,893	19,764	13,893	19,764	13,893
Adjusted *R*^2^	0.951	0.956	0.948	0.953	0.948	0.954
Store FE	Yes	Yes	Yes	Yes	Yes	Yes
Year-month FE	Yes	Yes	Yes	Yes	Yes	Yes
Other controls	Yes	Yes	Yes	Yes	Yes	Yes

****p* < 0.01, ***p* < 0.05, **p* < 0.1

Year-month fixed effects (FE) are unique for each year and month. A total of 32 year-month fixed effects were included in the regressions. Control variables for local temperature, gasoline price, CPI, and unemployment rate were included in the regressions but omitted in the table. Robust errors for coefficient estimates were clustered at the store level. The dataset, used in Columns (1), (3), and (5), included 625 stores in 159 cities and towns from five provinces including Ontario. The urban specifications Columns (2), (4), and (6) included 440 stores that are located in large urban areas, for instance, Toronto, ON. The analysis used observations from January 2017 to August 2019

^6^.

## Footnotes

1 It is worth nothing that many other risk-reduced substitutable products, such as snus, smokeless tobacco, heated tobacco, and pharmaceutical nicotine, were either not on the convenience store market during the study period or were strictly limited by regulatory policies.

2 A condition of being granted access to this data is that the identity of the convenience store chain must remain anonymous. JUUL Labs, Inc. does not own the data underpinning these analyses. Interested parties may contact Michael Fisher (Michael.fisher@juul.com) for additional information regarding these data.

3 We excluded data collected after August 2019 due to “E-cigarette and Vaping-Associated Lung Illness” (EVALI), an outbreak of illnesses strongly linked to use of illicit vaping products containing tetrahydrocannabinol-containing (THC) in the US (Centers for Disease Control and Prevention, February 25 2020) that received widespread news coverage in fall of 2019 and may have impacted use of ENDS products among adults who currently smoke (Dave et al. 2020).

4 In Columns (1) and (2), percentage effects are directly from the estimated coefficients. In Columns (3) to (6), the percentage effects are calculated by comparing to the monthly average from 09/2017 to 08/2018.

5 See National Health Service, Using e-cigarettes to stop smoking, available at https://www.nhs.uk/live-well/quit-smoking/using-e-cigarettes-to-stop-smoking/ (accessed March 16, 2023).

6 See Ministry of Health, Vaping Facts, available at https://vapingfacts.health.nz/ (accessed March 16, 2023).
